# Taking Ecological Function Seriously: Soil Microbial Communities Can Obviate Allelopathic Effects of Released Metabolites

**DOI:** 10.1371/journal.pone.0004700

**Published:** 2009-03-11

**Authors:** Harleen Kaur, Rajwant Kaur, Surinder Kaur, Ian T. Baldwin, Inderjit

**Affiliations:** 1 Max-Planck-Institute for Chemical Ecology, Department of Molecular Ecology, Jena, Germany; 2 Centre for Environmental Management of Degraded Ecosystems (CEMDE), University of Delhi, Delhi, India; 3 Botany Department, S.G.T.B Khalsa College, University of Delhi, Delhi, India; Massachusetts General Hospital, United States of America

## Abstract

**Background:**

Allelopathy (negative, plant-plant chemical interactions) has been largely studied as an autecological process, often assuming simplistic associations between pairs of isolated species. The growth inhibition of a species in filter paper bioassay enriched with a single chemical is commonly interpreted as evidence of an allelopathic interaction, but for some of these putative examples of allelopathy, the results have not been verifiable in more natural settings with plants growing in soil.

**Methodology/Principal findings:**

On the basis of filter paper bioassay, a recent study established allelopathic effects of m-tyrosine, a component of root exudates of *Festuca rubra* ssp. *commutata*. We re-examined the allelopathic effects of m-tyrosine to understand its dynamics in soil environment. Allelopathic potential of m-tyrosine with filter paper and soil (non-sterile or sterile) bioassays was studied using *Lactuca sativa*, *Phalaris minor* and *Bambusa arundinacea* as assay species. Experimental application of m-tyrosine to non-sterile and sterile soil revealed the impact of soil microbial communities in determining the soil concentration of m-tyrosine and growth responses.

**Conclusions/Significance:**

Here, we show that the allelopathic effects of m-tyrosine, which could be seen in sterilized soil with particular plant species were significantly diminished when non-sterile soil was used, which points to an important role for rhizosphere-specific and bulk soil microbial activity in determining the outcome of this allelopathic interaction. Our data show that the amounts of m-tyrosine required for root growth inhibition were higher than what would normally be found in *F. rubra* ssp. *commutata* rhizosphere. We hope that our study will motivate researchers to integrate the role of soil microbial communities in bioassays in allelopathic research so that its importance in plant-plant competitive interactions can be thoroughly evaluated.

## Introduction

**Figure 1:**
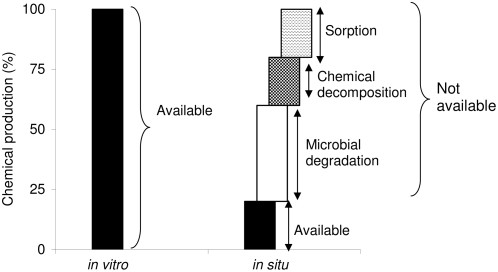
Schematic depiction of the differences in the availability/recovery of a putative chemical to a competing species from a given amount of chemical produced and released from a plant in *in vitro* assays as compared to *in situ* assays that include soil and the associated microbial (bulk and rhizosphere) communities. Sorption of chemicals onto soil particles, chemical decomposition and/or microbial degradation of chemicals are major mechanisms that influence their ability to accumulate to phytotoxic levels and influence the growth of neighboring target plants [13], [40], [41]. Sometimes physical sorption of a chemical on soil particles can actually concentrate the chemical to a level that may become physiologically active. Therefore, sorption may affect allelopathy both negatively and positively.

One of the fascinating but controversial processes in plant ecology is the mediation of competitive interactions among plants by plant-released metabolites, a mechanism referred to as “allelopathy.” In spite of the established role of soil microbial communities in interfering with the allelopathic activity of a metabolite [1]–[4], most studies use bioassays that do not incorporate a microbial component in their experimental designs. The high availability of a chemical to target species in *in vitro* bioassays may decrease to non-phytotoxic levels after its entry into the soil (Fig. 1). The negative effect on the growth of a plant of a certain species in a filter paper bioassay enriched with a single chemical is frequently used as evidence for an allelopathic interaction; we argue here that such evidence is insufficient and consider three recent examples: (±)-catechin, 8-hydroxyquinoline, and m-tyrosine.

The phytotoxic activity of (±)-catechin, a flavonoid exuded from roots of *Centaurea maculosa*, was initially established in experiments that used Murashige and Skoog (MS) basal medium [5], [6]. However, the role of catechin as an allelopathic agent became controversial when attempts were made to quantify catechin in soil [7]–[10]. Of 402 soil samples from 11 *C. maculosa* sites during two growing seasons, catechin (0.65±0.45 mg/g) was detected only from 20 samples collected from one site at one sampling time [11]. Although no empirical evidence is available to explain the lower levels of catechin, it was speculated that soil chemical properties and microbial communities could influence catechin accumulations in soil [8], [11]. When added at the levels of 340, 680 or 1020 µg/g soil, catechin recovery from soil was 0.4, 2.1 or 3.6%, respectively [12]. Inderjit et al. [12] showed that soil rich in organic matter made catechin toxic at concentrations (e.g., 266 or 400 µg/g soil) which otherwise were not toxic when the soil had a low organic matter content. While much more work is required to understand the role of organic matter in reducing the phytoxicity of catechin, one interesting possibility is sorption of the allelochemical to organic matter which in turn prevents their metal-mediated decomposition [13], a mechanism which may be particularly relevant for the next example.

Phytotoxicity of 8-hydroxyquinoline, a root-exuded metabolite from *Centaurea diffusa*, was also established using MS basal medium in Petri dishes [14] but Norton et al. [15] could not detect 8-hydroxyquinoline from soil in which *C. diffusa* had been growing in either the field or glasshouse. Since 8-hydroxyquinoline has affinity for cations such as aluminum, iron or magnesium, the addition of EDTA (disodium ethylenediaminetetraacetate) to the extraction solution improved the recovery of 8-hydroxyquinoline probably due to dissociation of 8-hydroxyquinoline-metal cation complex [15]. In a recent study, Tharayil et al. [16] examined the role of cations in determining the phytotoxicity of 8-hydroxyquinoline using filter paper bioassays and found that a binary mixture of 8-hydroxyquinoline and cations (particularly Fe or Cu) significantly reduced the phytotoxicity of 8-hydroxyquinoline. Since data are not available on the cation concentrations of *C. diffusa*-invaded soil and since the influence of microbial communities were excluded from this work, it remains an open question whether 8-hydroxyquinoline is allelopathic in soil.

On the basis of filter paper and agar bioassays, Bertin and colleagues [17] established the allelopathic potential of m-tyrosine, a metabolite exuded by the roots of *Festuca rubra* ssp. *commutata*. These results were extrapolated to the development of a biorational approach for weed control [18]. Here we show that, once again, the use of filter paper bioassays is not sufficient to evaluate the allelopathic potential of an exuded metabolite.

## Materials and Methods

### Filter paper bioassays

We tested the phytotoxic effects of m-tyrosine on lettuce (*Lactuca sativa*), littleseed canarygrass (*Phalaris minor*) and bamboo (*Bambusa arundinacea*) using filter paper and soil (non-sterile or sterile) bioassays. Since m-tyrosine was suggested to be a potent allelopathic and herbicidal compound [17], [18], we have chosen lettuce - a sensitive species widely used as an assay species in allelopathic research [19], bamboo - a native species of Indian subcontinent grown throughout the world [20], and littleseed canarygrass - an exotic weed found in wheat fields of northwestern India [21]. A 2500 µM aqueous stock solution of m-tyrosine was prepared, which was serially diluted to obtain 1250, 625, 312.5, 156.3 and 78.2 µM m-tyrosine (hereafter referred as 2500, 1250, 625, 313, 156 and 78 µM, respectively). For each species, 10 seeds were placed on Whatman # 1 filter paper in 9-cm Petri dishes. To maintain uniform moisture in the Petri dishes, a cotton pad was placed below the filter paper. In each Petri dish, 10 mL of each concentration of m-tyrosine or water (served as control) was added. Petri dishes were kept at 22 (for lettuce and littleseed canarygrass) or 30 (for bamboo) °C as per the specific growth requirements of the assay species. Each treatment was replicated five times. Root length of seedlings was measured after 7 d of placing seeds in control or m-tyrosine treatments. Treatments were compared for each species using one-way ANOVA and post ANOVA Tukey tests [22].

### Soil bioassays

Since soil microbes could degrade chemicals after their entry into soil [3], we studied the growth responses of different assay species in sterile and non-sterile soil amended with m-tyrosine. Soil sterilization has recently been used as a tool to establish the role of microbial communities in determining allelopathic effects [23]. Soil was autoclaved three successive times (120°C, 103 kPa pressure for 30 min). A 50 g non-sterile or sterile soil was treated with 15 mL of 0, 78, 156, 313, 625, 1250 or 2500 µM m-tyrosine to obtain final concentrations of 0, 4.25, 8.5, 17, 34, 68 or 136 µg m-tyrosine/g soil, respectively. Ten seeds of lettuce, littleseed canarygrass or bamboo were sown on 50 g m-tyrosine-treated and control soil. Each treatment was replicated 5 times. Data on root length were collected 7 d (for non-sterile soil) or 8 d (for sterile soil) after sowing. Due to the specific temperature requirements of lettuce and littleseed canarygrass, these species were grown at 22°C, while bamboo was grown at 30°C. Growth response of each assay species in m-tyrosine-treated soil and control soil were compared using one-way ANOVA and post ANOVA Tukey tests [22].

### m-Tyrosine recovery in soil

To examine the effect of rhizosphere-specific microbial activity on the availability of m-tyrosine, we studied m-tyrosine recovery from non-sterile soil with or without assay species at 22°C (for littleseed canarygrass and lettuce) or 30°C (for bamboo). Three seeds of lettuce, littleseed canarygrass or bamboo were sown in 10 g non-sterile soil and inoculated at 22 (lettuce and littleseed canarygrass) or 30°C (bamboo). After 8 d of seed germination, the non-sterile soil was treated with 0, 4.25, 8.5, 17, 34, 68 or 136 µg m-tyrosine/g soil and incubated under same conditions for 24 h. In order to investigate the recovery of m-tyrosine in soil devoid of microbes, sterile and non-sterile soils without assay species were treated with 0, 4.25, 8.5, 17, 34, 68 or 136 µg m-tyrosine/g soil and incubated at 22 or 30°C for 24 h. Each m-tyrosine treatment was replicated thrice.

After an incubation period of 24 h, soil samples were extracted in 5 ml water, and briefly mixed by vigorous vortexing. After centrifugation for 10 min at 4500 rpm, supernatant was passed through 0.2 µm filters (Sartorius) and placed in sample vials for high-pressure liquid chromatography (HPLC) analysis. m-Tyrosine recovery was measured by HPLC with an Agilent-HPLC, 1100 series equipped with an ODS inertsil C-18 column (3 µm, 150×4.6 mm i.d.) protected by a Phenomenex Security Guard C18 pre-column. The mobile phases were (A) water: 0.05% formic acid and (B) acetonitrile: 0.05% formic acid. The elution system was as follows: 0–3 min, 3% of B; 3–12 min, 3–50% of B; 12–15 min, 50% of B. The flow rate was 1 mL/min, the injection volume was 10 µL, and the column oven was set at 24°C, and the elutent was monitored at 280 nm. m-Tyrosine concentration among or between treatments was analyzed by means of one-way ANOVAs or t-tests, respectively [22].

In order to examine the root exudation of m-tyrosine in non-sterile soil, seeds (0, 3, 6 or 9 seeds/Petri dish) of *F. rubra* ssp. *commutata* were sown in 10 g soil at 22°C. Eight days after the germination of seeds, seedlings were removed, and soil was extracted with 10 mL water. Soil extracts were then subjected to HPLC as described above.

### Soil microbial activity

Measuring soil CO_2_ respiration by chemical titration is an effective method to compare microbial activity in different soils [24]. Three 5-cm Petri dishes containing 10 mL of 0.1 N NaOH were placed in the larger, 20-cm Petri dish filled with 250 g soil. Non-sterile soil was then treated with m-tyrosine to get a concentration of 0, 4.25, 8.5, 17, 34, 68 or 136 µg m-tyrosine/g soil. The 20-cm Petri dishes were then covered and sealed to avoid any loss of CO_2_. Three replicates were used. Soil was incubated at 22°C or 30°C, respectively for 24 h, and was terminated by adding 1 mL of 0.1 N BaCl_2_ to NaOH. A 10 mL of NaOH taken from blank, control or treatment was titrated against 0.1 N HCl, and amount of CO_2_ released was calculated [25].

## Results

### Growth bioassays

**Figure 2:**
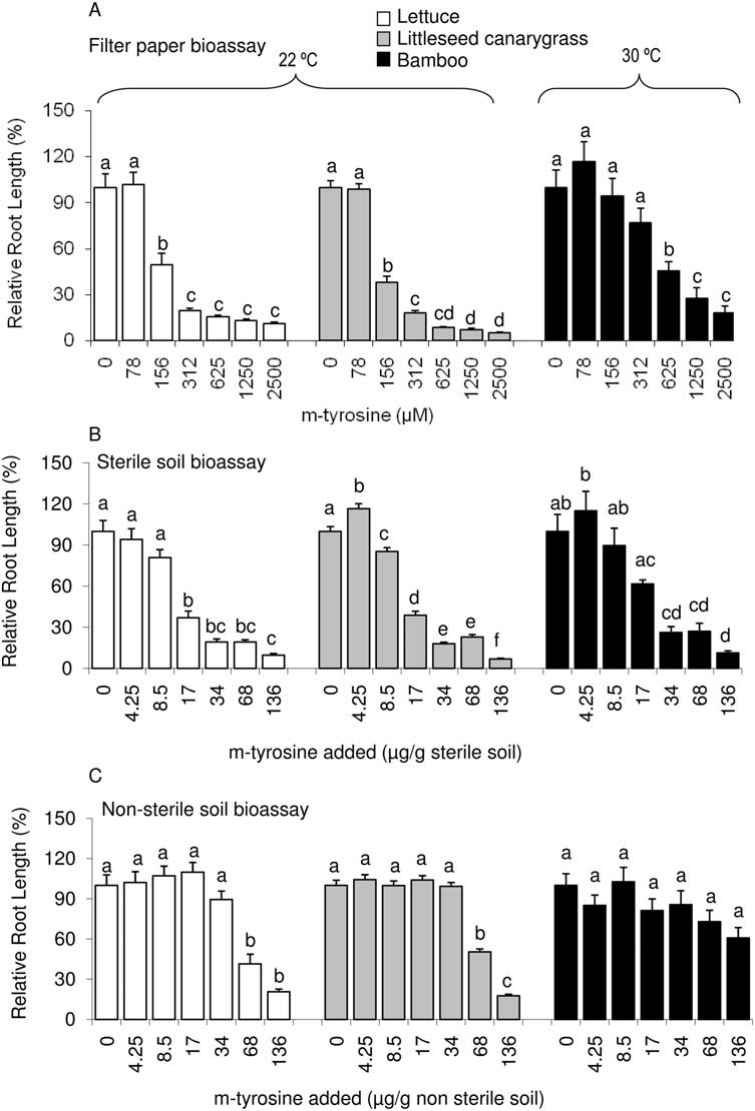
Relative root length (%) of lettuce, littleseed canarygrass and bamboo when exposed to 0, 78, 156, 313, 625, 1250 or 2500 µM m-tyrosine in filter paper bioassays (upper panel), and when grown in sterile (middle panel) or non-sterile (lower panel) soil treated with 0, 4.25, 8.5, 17, 34, 68 or 136 µg m-tyrosine/g soil. Root length of 3 assay species in soil treated with 0 µg m-tyrosine/g soil is taken as 100%. The root lengths of respective assay species were then calculated relative to the zero level treatment of m-tyrosine. Error bars represent 1 SE and shared letters indicate no significant differences among treatments and control as determined by one-way ANOVA with treatment as fixed variable, and post ANOVA Tukey test (P<0.05).

Root growth of all the three assay species was suppressed when grown either on filter paper moistened with m-tyrosine (F_lettuce_ = 56.65, df = 6, 284; P<0.001; F_littleseed canarygrass_ = 301.1; df = 6, 318; P<0.001; F_bamboo_ = 16.97, df = 6, 230, P<0.001) or in sterile soil treated with m-tyrosine (F_lettuce_ = 50.1; df = 6, 216; P<0.001; F_littleseed canarygrass_ = 290.7; df = 6, 258, P<0.001; F_bamboo_ = 16.58; df = 6, 237; P<0.001) (Fig. 2 upper and middle panel). In non-sterile soil bioassays, root growth of littleseed canarygrass (F_littleseed canarygrass_ = 149.27; df = 6, 260, P<0.001) and lettuce (F_lettuce_ = 26.819; df = 6, 201; P<0.001) was inhibited in soils amended with 68 or 136 µg/g tyrosine (Fig. 2 lower panel). Bamboo, however, was not sensitive to the m-tyrosine treatments (F_bamboo_ = 2.138; df = 6, 197; non-significant) (Fig. 2 lower panel).

### m-Tyrosine recovery in soil

**Figure 3:**
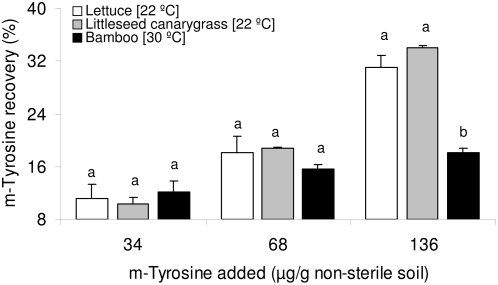
m-Tyrosine recovery (%) from m-tyrosine treated non-sterile soil with seedlings of lettuce, littleseed canarygrass or bamboo. Shared letters indicate no significant differences in mean m-tyrosine recovery from the soils of the three assay species as determined by one-way ANOVAs, and post ANOVA Tukey test (P<0.05).

m-Tyrosine was not detected in non-sterile soil amended with 4.25, 8.5 or 17 µg m-tyrosine/g soil with any of the assay species (data not shown). No significant difference in m-tyrosine recovery was observed among three assay species at 34 or 68 µg m-tyrosine/g soil (Fig. 3). However, in soil treated with 136 µg m-tyrosine/g soil, a significantly lower recovery of the m-tyrosine was observed with bamboo (18.1%) as compared to lettuce (31.1%) or littleseed canarygrass (34.1%) (F = 24.50, df = 2, 6; P<0.001; Fig. 3).

**Figure 4:**
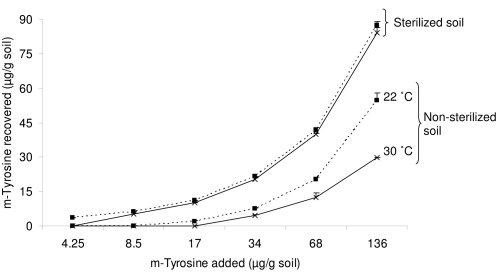
m-Tyrosine (µg/g soil) recovery from non-sterile or sterile soil treated with 0, 4.25, 8.5, 17, 34, 68 or 136 µg m-tyrosine/g soil and incubated at 22 or 30°C. Error bars indicate 1 SE.

We also examined the recovery of m-tyrosine in sterile and non-sterile soil without assay species when treated with 0, 4.25, 8.5, 17, 34, 68 or 136 µg m-tyrosine/g soil and incubated at 22 or 30°C. A higher availability of m-tyrosine was observed in non-sterile soil, incubated at 22°C, treated with 17 (12.1%), 34 (22.1%), 68 (29.7%) or 136 (40.3%) µg m-tyrosine/g non-sterile soil compared to non-sterile soil, incubated at 30°C, treated with 17 (0%), 34 (13.1%), 68 (18.5%) or 136 (21.8%) µg m-tyrosine/g non-sterile soil (Fig. 4). The recovery of m-tyrosine in sterile soil incubated at 22°C when treated with 8.5, 17, 34, 68 or 136 µg m-tyrosine/g soil was 73.2, 64.1, 63.2, 61.2 or 64.3%, respectively; which was statistically similar to recoveries (62.7, 59.5, 60.1, 58.6 or 61.8%, respectively) observed in sterile soil incubated at 30°C (t-test, P<0.05).

### Soil microbial activity

**Figure 5:**
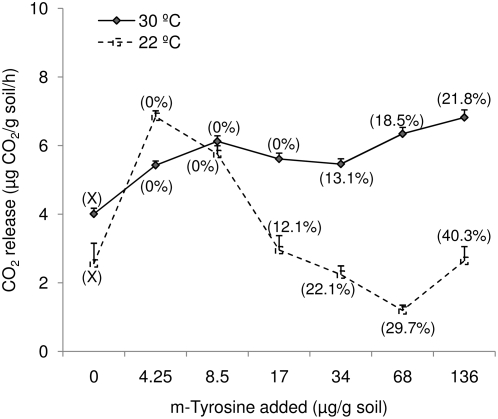
Microbial activity as indicated by CO_2_ release (µg CO_2_ released/g soil/h) of soil treated with 0, 4.25, 8.5, 17, 34, 68 or 136 µg m-tyrosine/g soil, incubated at 22 or 30°C. Error bars indicate 1 SE. Values in parenthesis indicate % recovery of m-tyrosine in soil.

Soil not treated with m-tyrosine when incubated at 30°C had higher CO_2_ release (36.05%; t = 2.338; P_(2-tailed)_ = 0.035) compared to untreated soil incubated at 22°C (Fig. 5). The CO_2_ release in treated soil incubated at 30°C was significantly (P<0.0001) higher compared to untreated soil incubated at 30°C. No significant differences (except 4.25 or 8.5 µg m-tyrosine/g soil) were observed in the CO_2_ released from m-tyrosine-treated soil incubated at 22°C compared to untreated soil incubated at 22°C. A significantly higher CO_2_ release from soil treated with 17 (t = 5.745; P_(2-tailed)_<0.0001), 34 (t = 10.625; P_(2-tailed)_<0.0001), 68 (t = 20.660; P_(2-tailed)_<0.0001) or 136 (t = 8.397; P_(2-tailed)_<0.0001) µg m-tyrosine/g soil was observed at 30°C compared to respective soils incubated at 22°C (Fig. 5).

## Discussion

In the filter paper bioassays, m-tyrosine inhibited the root growth of lettuce and littleseed canarygrass at 156 µM or above, while with bamboo higher concentrations were required and root growth was significantly suppressed only at 625 µM or above (Fig. 2 upper panel). These results are consistent with the observations of Bertin et al. [17]: m-tyrosine is phytotoxic to a number of other plant species in filter paper bioassays. However, filter paper bioassays can overestimate the residence time of exuded chemicals in the rhizosphere and the ability of a chemical to influence the growth of neighboring plants.

Results similar to those obtained from the filter paper bioassays were found with bioassays using sterile soil (Fig. 2 middle panel). However, when non-sterile soil was used, evidence for the phytotoxicity of m-tyrosine vanished for bamboo and growth inhibition of lettuce or littleseed canarygrass was only observed at the two highest concentrations tested (Fig. 2, lower panel). Root growth was only suppressed when lettuce and littleseed canarygrass were grown in non-sterile soil amended with 68 or 136 µg m-tyrosine/g soil.

The lack of m-tyrosine phytotoxicity at 136 µg m-tyrosine/g soil was associated with its lower recovery (18.1%) in non-sterile soil with bamboo compared to non-sterile soil with lettuce (31.1%) or littleseed canarygrass (34.1%) (Fig. 3). The lower recovery of m-tyrosine in bamboo soil could be due to either temperature-dependent non-microbial degradation, temperature-dependent microbial degradation or rhizosphere-specific microbial activity in bamboo rhizosphere. The lack of significant differences in m-tyrosine recovery from sterile soil at 22 or 30°C ruled out temperature-dependent non-microbial degradation of m-tyrosine (Fig. 4).

Since measuring soil CO_2_ efflux is a reliable technique for comparing microbial activity in different soils [24], we measured CO_2_ release in untreated and m-tyrosine-treated soil incubated at 22 or 30°C. We observed a higher CO_2_ release in the untreated soil incubated at 30°C (4±0.2 µg CO_2_ released/g soil/h) compared to 22°C (2.6±0.6 µg CO_2_ released/g soil/h). A higher m-tyrosine recovery of 12.1, 22.1, 29.7 and 40.3% was observed in non-sterile soil treated with 17, 34, 68 or 136 µg m-tyrosine/g soil at 22°C compared to 0, 13.1, 18.5 or 21.8%, respectively in m-tyrosine treated soil at 30°C. The lower recovery was associated with higher CO_2_ efflux suggesting higher microbial activity in soil treated with 17 (47.4%), 34 (59.1%), 68 (81.1%) or 136 (61.4%) µg m-tyrosine/g soil at 30°C compared to respective soils incubated at 22°C (Fig. 5) as others have found [26]. However, the effects were not strictly concentration-dependent (Fig. 5). When incubated at 22°C, soil treated with 4.25 µg m-tyrosine/g had a higher CO_2_ release (except from the soil treated with 8.5 µg m-tyrosine/g soil) compared to soil treated with 17, 34, 68 or 136 µg m-tyrosine/g soil at 22°C. No differences in the CO_2_ release were observed among soils treated with 17, 34, 68 or 136 µg m-tyrosine/g soil at 22°C (Fig. 5). Negative impacts of m-tyrosine on *Bacillus* sp. and *Escherichia coli* are known [27]–[29], which to some extent explain the dome-shaped curve for microbial activity at 22°C (Fig. 5). It may be possible that increased nitrogen availability in response to m-tyrosine addition allows particular soil bacteria to grow better at low m-tyrosine concentrations, but higher concentrations inhibit bacterial growth and CO_2_ release. This aspect, however, needs further experimental evidence. Higher microbial activity in soil incubated at 30°C likely accounts for the more rapid degradation of m-tyrosine and the disappearance of phytotoxic effects to bamboo. Since the recovery of m-tyrosine in non-sterile soils with and without bamboo did not differ (Fig. 4), the possibility that bamboo rhizosphere-specific microbial activity was involved is unlikely. When bamboo was grown in non-sterile soil treated with 34, 68 or 136 µg m-tyrosine/g soil, m-tyrosine recovery was 12.1, 15.6 or 18.1% respectively, compared to 13.1, 18.5 or 21.8% in non-sterile soil without bamboo. When non-sterile soil without littleseed canarygrass or lettuce was treated with 17, 34, 68 or 136 µg m-tyrosine/g soil, the recovery was 12.1, 22.1, 29.7 or 40.3%, respectively. However, the recoveries decreased when non-sterile soil with lettuce (0, 11.3, 18.1or 31.1%, respectively) or littleseed canarygrass (0, 10.3, 18.7 or 34.1%, respectively) were measured. Hence there is a possibility of rhizosphere-specific microbial breakdown in the rhizosphere of lettuce and littleseed canarygrass. Rhizospheres are known to harbor microbial populations specific to a particular cultivar [30] and this specificity should be incorporated in the allelopathy bioassays [31], [32].

We did not detect m-tyrosine in non-sterile soils with 3, 6 or 9 seedlings of *F. rubra* ssp. *commutata*. Bertin et al. [17] reported that *F. rubra* ssp. *commutata* releases a large amount of m-tyrosine into the rhizosphere, but the methods used could overestimate the release rates. These authors isolated m-tyrosine from the rhizosphere of *F. rubra* ssp. *commutata* after germinating its seeds between two layers of cheesecloth arranged on capillary mat system. Freshly harvested or intact roots were soaked in water and it is unclear how the pools of m-tyrosine extracted from roots (33–43% of dry weight of root exudate extract) reflect the amount actually released from the plant that could interact with competing plants. Moreover, it is difficult to collect root surface washings in a system without causing physical damage to the roots, which is unlikely to occur in the artificial soil system. In natural conditions, biotic stresses such as herbivory, however, might cause a local release of m-tyrosine. Ecologists have often criticized the extraction procedures used in past experiments claiming the demonstration of allelopathic effects. Harper [33, p 369] stated, “*Almost all species can, by appropriate digestion, extraction and concentration, be persuaded to yield a product that is toxic to one species or another*”. These comments hold for most of the recent claims of allelopathic function [5], [14], [16], [34], [35]. The large amounts of m-tyrosine contained in the roots of *F. rubra* ssp. *commutata* could have some ecological function, which yet remains to be discovered.

While m-tyrosine may exert allelopathic effects at high concentrations, our research suggests that the quantities of m-tyrosine released from the plant are unlikely to persist in soil to exert phytotoxic effects. Soil microbial communities form a major sink that influences accumulation of a metabolite to phytotoxic levels (Fig. 1). Microbial degradation of some important allelochemicals such as benzoxazinoids [36], juglone [37], [38], and several flavonoids including catechin, rutin and quercetin [4] are well established.

Bioassays for allelopathy should investigate rhizosphere-specific and bulk soil microbial activity; and also temperature-driven microbial activity. The differences between species that are ‘sensitive’ or ‘resistant’ to a putative allelochemical, as is commonly invoked in invasion ecology, may reflect differences in microbial communities harbored by different plant species. Although it is well known that *in vitro* demonstrations of allelopathic activity of a chemical can rarely be applied to field situation, the most recent studies published in high profile journals [5], [14], [16], [17], [39] still based their conclusions on *in vitro* experiments. It is our hope that this study will motivate researchers to integrate the role of soil microbial communities in bioassays in allelopathic research so that its importance in plant-plant competitive interactions can be thoroughly evaluated.
